# Challenges in the Detection of SARS-CoV-2: Evolution of the Lateral Flow Immunoassay as a Valuable Tool for Viral Diagnosis

**DOI:** 10.3390/bios12090728

**Published:** 2022-09-05

**Authors:** Nayeli Shantal Castrejón-Jiménez, Blanca Estela García-Pérez, Nydia Edith Reyes-Rodríguez, Vicente Vega-Sánchez, Víctor Manuel Martínez-Juárez, Juan Carlos Hernández-González

**Affiliations:** 1 Área Académica de Medicina Veterinaria y Zootecnia, Instituto de Ciencias Agropecuarias, Universidad Autónoma del Estado de Hidalgo, Av. Universidad km 1 Exhacienda de Aquetzalpa A.P. 32, Tulancingo 43600, Mexico; 2 Department of Microbiology, Instituto Politécnico Nacional, Escuela Nacional de Ciencias Biológicas Prolongación de Carpio y Plan de Ayala S/N, Col. Santo Tomás, México City 11340, Mexico

**Keywords:** lateral flow immunoassay, biosensors, COVID-19, SARS-CoV-2, diagnosis, antibodies

## Abstract

SARS-CoV-2 is an emerging infectious disease of zoonotic origin that caused the coronavirus disease in late 2019 and triggered a pandemic that has severely affected human health and caused millions of deaths. Early and massive diagnosis of SARS-CoV-2 infected patients is the key to preventing the spread of the virus and controlling the outbreak. Lateral flow immunoassays (LFIA) are the simplest biosensors. These devices are clinical diagnostic tools that can detect various analytes, including viruses and antibodies, with high sensitivity and specificity. This review summarizes the advantages, limitations, and evolution of LFIA during the SARS-CoV-2 pandemic and the challenges of improving these diagnostic devices.

## 1. Introduction

In December 2019, in Wuhan, China, a severe outbreak of acute respiratory illness caused by a novel beta-coronavirus identified as SARS-CoV-2 (severe acute respiratory syndrome associated coronavirus-2) was reported. The virus spread rapidly, and on 11 March 2020, the World Health Organization (WHO) declared COVID-19 (coronavirus disease-19) as a pandemic causing high severe morbidity and mortality worldwide. The reported cases globally exceed 596 million, and deaths are nearly 6.4 million as of 24 August 2022 [1].

Accurate diagnostic tests to identify the causative agent of COVID-19 are essential to prevent the spread of the virus in the population, provide prompt and timely treatment, and avoid future viral variants. The WHO recommends viral gene detection by reverse transcription-polymerase chain reaction (RT-PCR) as the gold standard for diagnosing a SARS-CoV-2 infection [2,3]. However, RT-PCR demands trained personnel, laboratory equipment, and expensive reagents, limiting its implementation in point-of-care testing (POC) in the field [4,5]. Moreover, inadequate collection of clinical specimens or poor handling of a sample during testing can result in false-negative RT-PCR [6].

The pandemic SARS-CoV-2 represents an unprecedented emergency globally, and its dissemination in the future leaves an uncertain path forward. The immunization expressed the hope for COVID-19 control and possible eradication [7]. The WHO reported that the new variant B.1.1.529, also known as omicron, has a deletion in the S gene, which can cause failure in some RT-PCR assays. Other PCR methods are proposed in these cases, such as Single Nucleotide Polymorphism (SNP) [8]. SNP assays enable the detection of single nucleotide changes within the SARS-CoV-2 genome to detect variants of concern as a complement to whole genome sequencing [8]. However, this diagnostic resource implies a high cost of specialized equipment and trained personnel.

Recent advances in nanotechnology and biotechnology have enabled the development of biosensors for disease diagnosis. A conventional biosensor device consists of a bio-receptor where an immobilized biological component is supported in nanomaterials, a site that recognizes the analyte in the sample. The transducer confers classification to a biosensor by the type of signal (electric current, thermal changes, magnetic fields, etc.), which sends analog data to the processing system that converts them into digital data [9]. The biosensors developed in the diagnosis of COVID-19, according to the target molecule identified in the sample, can detect high specificity antibodies in serum or high-affinity molecules of the SARS-CoV-2 virus [10,11,12]. Biosensor development for SARS-CoV-2 diagnostic is a challenge to researchers. Biosensors with transducers have demonstrated superior specificity and sensitivity to other diagnostic techniques, but only under controlled laboratory conditions (known virus concentration in buffered solution or hyperimmune sera produced in laboratory animals) [13,14]. Most of these devices show poor performance when tested in clinical practice due to contaminants abundant in enzymes in pharyngeal swabs, mucus, and cellular detritus, among others. The difficulties in clinical aspects are threshold (cut-off value), qualitative or quantitative reader, and others [15]. Sometimes, the material that makes up the biosensor is not easy to obtain and manipulate, as is the case of graphene, which requires nanotechnology facilities. These devices for SARS-CoV-2 diagnostic are in the experimental phase, and most are not commercially available.

**Figure 1:**
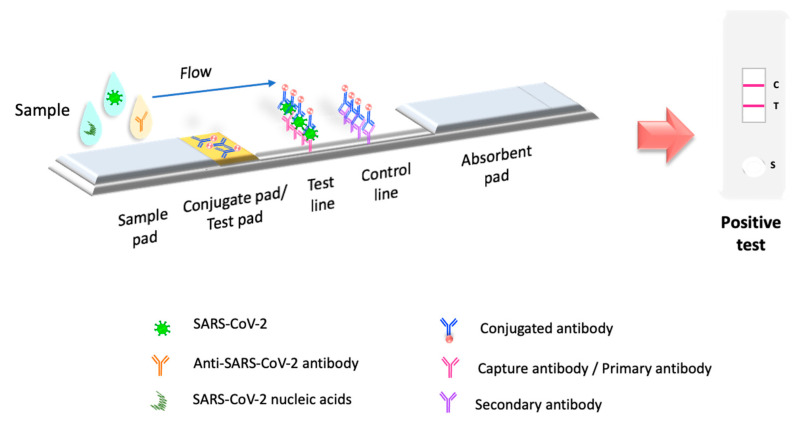
Principle of LFIA test. LFIA test detects the target molecule on an absorbent membrane with antibodies aligned to form the test and control lines. The sample is placed on a sample pad, then migrates to the conjugate pad, which contains the immobilized conjugate, usually made of nanoparticles (colloidal gold, colored or fluorescent latex, colored cellulose) conjugated to antibodies or antigens. The sample interacts with the conjugate, and both migrate to the next section of the strip, where the biological components of the assay (proteins/antibodies/antigens) are immobilized. In this section, the analyte and conjugate are captured. Excess reagent passes through the capture lines and accumulates on the absorbent pad. The results are interpreted on the nitrocellulose membrane as the presence or absence of the test and control lines.

Recent decades’ development of rapid test biosensors represents a remarkably effective tool for diagnosis, including SARS-CoV-2, as they are fast, sensitive, and inexpensive. The simplest biosensors (without transducer) are paper-based and do not require multiple steps or the addition of any solution other than the patient sample [16]. The lateral flow immunoassay (LFIA) is the most representative and commercially available among the different types of biosensors. LFIAs have been widely recognized as fast biosensors with point of care (POC) status [17,18,19]. LFIA test detects the target molecule on an absorbent membrane with antibodies aligned to form the test and control lines; the signal is analyzed qualitatively in a visual or semi-quantitative reading [18,20] (Figure 1). LFIA is a valuable tool that has been used for detecting, diagnosing, and monitoring various viruses such as human immunodeficiency virus (HIV), human adenovirus, influenza A H1N1 virus, and SARS-CoV-2 [21,22,23].

During the pandemic, LFIA has been used as a mass test of POC for early detection of SARS-CoV-2 globally, in contrast to RT-PCR testing [24]. LFIA diagnostic tests have a dual function: on the one hand, they can detect the patient’s antibodies to determine protection against SARS-CoV-2, and on the other hand, they can detect viral antigens for early diagnosis of infection, including asymptomatic people. This review analyzes the evolution of the LFIA biosensor for the early diagnosis of SARS-CoV-2, highlighting its improvements since the pandemic began.

## 2. SARS-CoV-2 Overview

Coronaviruses are a large family of well-established pathogens of various hosts, including domestic animals, wild animals, and humans [25]. Viruses that caused previous outbreaks in humans, causing severe respiratory illness, lung injury, and death, are SARS-CoV (severe acute respiratory syndrome coronavirus) in 2003 and MERS-CoV (Middle East respiratory syndrome coronavirus) in 2012 [26]. Recent genome analysis with various bioinformatics tools demonstrated that SARS-CoV-2 has a highly similar genome as the Bat coronavirus and receptor-binding domain (RBD) of spike glycoprotein as Malayan pangolin coronavirus [27]. This evidence indicates that the horseshoe bat is the natural reservoir, and primary evidence suggests that the Malayan pangolin is an intermediate host [27].

SARS-CoV-2 is an enveloped virus with a positive-sense single-stranded RNA. The genome size of this pathogen varies from 29.8 kb to 29.9 kb [28]. The virus encodes at least 29 proteins. The structural proteins are spike (S), membrane (M), envelope (E), and nucleocapsid (NP) proteins [29]. The non-structural proteins (nsps) have functions required for replication and transcription in the virus life cycle [30]. The viral particle size ranges from 80 to 120 nm [31].

The mechanism of viral infection in humans is through droplets and aerosols, which can travel through the air [32]. Infection occurs in cells that express ACE2 (angiotensin-converting enzyme 2) and TMPRSS2 (transmembrane serine protease 2) [33]. The coronavirus S protein binds to the ACE2, the primary receptor for SARS-CoV-2 that mediates virus entry into cells, and TMPRSS2 cleaves the S protein (into S1 and S2 subunits) of SARS-CoV-2 facilitating the fusion of SARS-CoV-2 and cellular membrane [33,34,35]. Moreover, it has been demonstrated that the endosomal cysteine proteases cathepsin B and cathepsin L can also contribute to this process [34,36,37]. In the respiratory tract, ACE2 and TMPRSS2 are expressed in the secretory and ciliated cells in the nose, secretory and ciliated cells in the conductive airways, in the type II alveolar cells in the lung as well as in corneal conjunctiva in the eye [38,39,40,41].

The etiological virus of the pandemic has continuously evolved, with many variants emerging worldwide. Variants are categorized as the variant of interest, variant of concern, and variant under monitoring [42]. There are five SARS-CoV-2 lineages designated as the variant of concern alpha, beta, gamma, delta, and omicron variants [43]. These variants increase transmissibility compared to the original virus and potentially increase disease severity [44].

## 3. Immune Response against SARS-CoV-2 in Brief

The SARS-CoV-2 infection involves diverse stages in the individual: start of infection, disease development, recovery, or systemic compromise. Each infection stage triggers and modulates innate and adaptative immune system mechanisms. Although SARS-CoV-2 is a virus that humanity is learning about, the immune response is equipped with mechanisms capable of dealing with this new threat. In the initial phase of SARS-CoV-2 infection, the individual presents a presymptomatic phase lasting up to 5 days, in which a high viral load is present [45]. In these early days of infection, antibodies may not have been produced. Therefore, innate immunity is the first activated. The innate immune response comprises soluble and cellular components that respond nonspecifically against the virus. The cellular compounds include dendritic cells (DC), monocytes, macrophages, neutrophils, natural killer (NK) cells, and other innate lymphoid cells (ILCs) [46]. Whereas soluble components include complement systems, soluble proteins, interferons, chemokines, and naturally occurring antibodies [47]. Immune response cells recognize pathogen-associated molecular patterns (PAMPs) of SARS-CoV-2 through pattern recognition receptors (PRRs) such as Toll-like receptors (TLR), RIG-I-like receptors (RLR), and melanoma differentiation-associated protein 5 (MDA5). The viral sensing triggers the activation of signaling pathways which induce the production of immune mediators to generate an antiviral state mainly mediated by type I (IFN-α/β) and type III (IFN-λ) interferons (IFNs) [48]. Reports have described that robust IFNs production during the early stage of infection is required to have a protective innate immune response against the virus [49]. On the contrary, an inadequate and slow response to type I and type III IFNs due to virus evasion mechanisms, host comorbidities, or genetic defects cause an exacerbated immune response. This inadequate response induces elevated levels of chemokines (CCL2, CCL8, CXCL2, CXCL8, CXCL9, and CXCL16), high expression of proinflammatory cytokines such as IL-6, IL-10, IL-1, and TNF, in addition to activation, and recruitment of immune cells [50,51]. The called “cytokine storm” leads to unbalanced levels of proinflammatory and antiviral mediators that remain the leading cause of ARDS and multi-organ failure [49,50,52].

On the other hand, the adaptive immune response is orchestrated by CD8+ T lymphocytes, TCD4+, and B lymphocytes, responsible for immunological memory. In response to SARS-CoV-2 infection, it has been shown that non-severe patients or patients with mild symptoms have a low viral load and may not have produced antibodies [53,54]. In contrast, antibodies have been detected by immunoassay tests and biosensors in patients with severe symptoms or cases [53,55]. Patients with a high viral load activate the humoral immune response in the first two weeks of infection [56]. The first seroconversion of antibodies is against protein N, followed by protein S of SARS-CoV-2 in patients with disease symptoms [57]. Immunoglobulins IgA and IgM begin to be detected within the first ten days of infection; however, both antibodies can cross-react with protein N, which is highly conserved among coronaviruses [58,59]. Moreover, high levels of IgG1 and IgG3 are expressed ten to fourteen days after infection in patients with disease symptoms [60,61]. Older adults and seriously ill individuals reach high specificity antibodies concentrations against SARS-CoV-2 S protein.

Due to the urgency of reducing thousands of people’s cases and deaths, scientists have developed several vaccines against COVID-19. Efforts are being made to apply vaccines with emergency use authorization to the world population. Vaccination elicits immune responses capable of potently neutralizing SARS-CoV-2. However, the available data show that most approved COVID-19 vaccines protect against severe disease but do not prevent the clinical manifestation of COVID-19 [62]. Instead, it has been demonstrated that new variants with mutations in the spike, the main target of neutralizing antibodies, can escape the neutralization of humoral immunity [63,64].

## 4. SARS-CoV-2 Detection

Molecular tests or biosensors are the tools for detecting SARS-CoV-2 nucleic acids/ antigens/antibodies against the virus (Figure 1). In the early part of the illness, viral particles and their subunits can be detected; beyond the first two weeks of illness onset, antibodies against the virus could be detected [65]. The SARS-CoV-2 infection stage is highly correlated to the diagnostic technique recommended for the pandemic. Early diagnosis of the disease and isolation of infected people is key to controlling the transmission of SARS-CoV-2 [66,67]. In the initial phase of SARS-CoV-2 infection, the individual presents a presymptomatic phase lasting up to 5 days, in which a high viral load is present [45]. During these early days of infection, antibodies may not be detected. Therefore, since the pandemic began, the diagnostic method has been based on detecting viral genes using the molecular PCR technique, the gold standard worldwide [2,3,68]. The pandemic has exceeded the ability to identify the virus in laboratories using molecular techniques; this has motivated the development of new technologies for the rapid detection of SARS-CoV-2 that are easy to perform compared to molecular tests in clinical laboratories. LFIA has been the unique device approved and available to use in mass worldwide. Biosensors with transducers are developing in SARS-CoV-2 diagnostic. However, most nanomaterials used in these biosensors present interferences with contaminants in human samples compared to performance under experimental conditions. It is important to emphasize that LFIAs have the unique properties of availability, accessibility, economy, and POC (including home use), these characteristics that are not shared by all biosensors with a transducer. In addition, biosensors with transducers require exclusive handling in laboratories certified under the Clinical Laboratory Improvement Amendments of 1998 [69,70]. The FDA have to date approved only one piezoelectric biosensor [69] (Figure 1).

## 5. Lateral Flow Immunoassay Evolution in the Pandemic

LFIAs are devices with features designed into POC testing technologies that fulfill ASSURED criteria (affordable, sensitive, specific, user-friendly, rapid and robust, equipment-free, deliverable to end-user) [71]. LFIAs have been widely used to diagnose bacterial and viral infections, including SARS-CoV-2. At the beginning of the pandemic, in North America and Europe, patients with symptoms consistent with COVID-19 but negative for SARS-CoV-2 by RT-PCR could be diagnosed by serological testing using LFIA [72]. Furthermore, these rapid detection tests were approved for emergency to detect serum antibodies in symptomatic patients [69]. However, the start of vaccination stimulated the production of protective antibodies in the world population, so the LFIA that detected antibodies became obsolete for early diagnosis of the disease.

Only LFIAs that detect viral antigens have been approved worldwide for the diagnosis of COVID-19 [69,73]. The manufacture of LFIAs continues to be developed to increase their sensitivity and specificity, implementing nanomaterials for their manufacture and advanced technology in chromatography. The future of these devices makes them as efficient as biosensors in diagnosing SARS-CoV-2, maintaining their application as a complementary test to PCR.

### 5.1. Lateral Flow Immunoassay Antibody Testing in COVID-19 Pandemic

**Figure 2:**
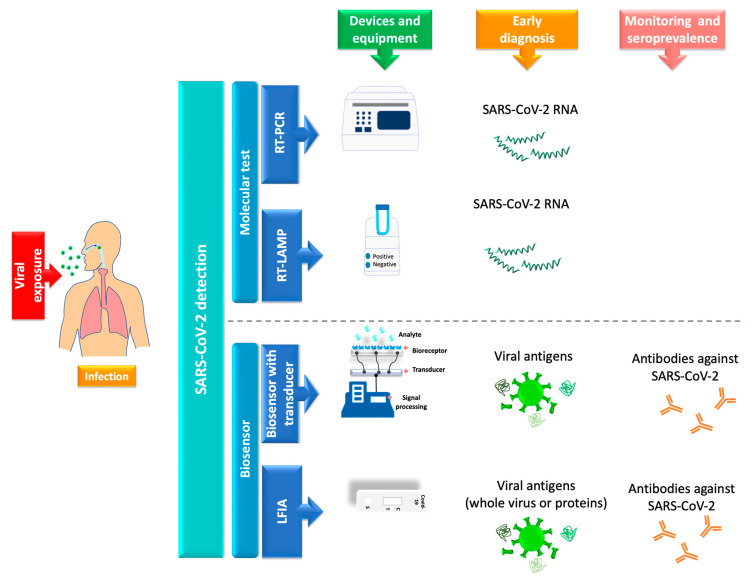
Main methods for detecting SARS-CoV-2. Currently, the detection of SARS-CoV-2 is carried out using molecular tests and biosensors. RT-PCR is the gold standard for detecting SARS-CoV-2 viral RNA. There are other available molecular tests used based on RT-LAMP. Biosensors with transducers have been developed to detect viral antigens or antibodies. LFIA is the representative test for mass diagnosis of SARS-CoV-2 in POC and antibody prevalence studies worldwide. These biosensors have been widely marketed because they can be applied at the POC and are inexpensive, fast, and easy to read.

At the beginning of 2020, LFIAs biosensors were commercially available to be used in mass to detect IgM/IgG antibodies against the new SARS-CoV-2 virus [3]. These devices were recommended in patients with clinical symptoms of SARS-CoV-2. Their main advantages were used at the POC, with qualitative outcomes in just 15 min or less, reaching a larger population without saturating the capacity of laboratories [74]. The pressing need to diagnose COVID-19 rushed manufacturing of large-scale, accurate, and affordable diagnostic immunoassays, including LFIA [69] (Figure 2). As of 2020, after evaluating five immunoassays and one lateral flow immunochromatography for anti-SARS-CoV-2 antibodies detection, FDA-EUA (Food and Drug Administration—Emergency Use Authorization) approved only two manufacturers of LFIAs to detect IgG/IgM anti-SARS-CoV-2 [3]. At the moment, forty-eight LFIAs manufacturing laboratories were granted EUA authorization to apply for SARS-CoV-2 diagnostic [69]. The marketing was exceptionally authorized without prior evaluation in the interest of world public health. Whereas the pandemic started, LFIAs tests showed variable performance when assessing test sensitivity and specificity towards the main immunogenic structures of the virus. In pediatric age (2 months to 18 years), COVID-19 symptoms are generally less severe than in adults, and a low level of antibodies is produced [75]. This immune response is associated with a low yield of sensitivity of LFIA antibody detection (about 70%); this value was lower in the second week after disease onset. [76]. In England, LFIA was applied twice per week to the general population, including asymptomatic individuals. False-positive results caused unnecessary isolation involving the cost of shutting down entire economic sectors [77].

#### LFIA Is a Quantitative Tool for Detecting Antibodies against SARS-CoV-2

Serological tests detect the presence of antibodies in the blood from the adaptive immune response to an infection. LFIAs as diagnostic serological tests for COVID-19, are designed to absorb a blood drop sample obtained by puncture onto a nitrocellulose membrane that captures and detects IgG antibodies or IgM/IgG antibodies [78,79,80,81]. The result is visualized by precipitation of usually gold nanoparticles or other colored nanoparticles to generate colored lines on the membrane [82]. Early in the pandemic, the FDA granted emergency clearance to LFIAs that detect IgG/IgM antibodies to diagnose COVID-19. Some of these tests did not meet the clinical serologic performance estimates required to meet EUA efficacy and risk/benefit standards, so they were revoked [3]. This evidence contributed to the withdrawal of most LFIA antibody screening tests for the diagnosis of COVID-19.

In 2021, the need for LFIAs antibody tests to detect with high sensitivity and/or quantitative mode neutralizing antibodies in individuals immunized or recovered from natural COVID-19 illness resurfaced [83,84,85]. The novel vaccines manufactured against SARS-CoV-2 make it necessary to assess the efficiency of immunization to produce neutralizing antibodies against SARS-CoV-2 and how long they last in the vaccinated individual [84,86]. The speed and affordability of the LFIA test allow timely detection of individuals who show a short or absent humoral immune response. On the other hand, in 2020, the USA approved an emergency authorization to use plasma therapy as part of treatment in patients with COVID-19 [87]. This process consists of obtaining convalescent plasma from patients recovered from COVID-19, abundant in neutralizing antibodies, to treat patients. [88]. LFIA is an effective tool in both cases because of its speed and lack of laboratory equipment requirements. LFIAs have shifted from COVID-19 diagnosis to monitoring post-infection or post-vaccination antibody production in the global population. However, LFIA tests had to improve sensitivity and a positive cut-off threshold limit for post-vaccination antibody detection and/or convalescent patients. COVID-19 being a newly emerging disease, it was necessary to determine the exact disease stage in which the LFIA antibody tests should be applied to the global population. It was necessary to analyze the humoral immune response in the world population at different stages of the disease (uninfected, asymptomatic, symptomatic, convalescent, and reinfected) [89,90,91,92]. Other factors such as age, sex, comorbidities, or high risk in healthcare personnel are involved in the variations of the humoral response [93]. On the other hand, the manufacture of LFIAs focused its development on the incorporation of new nanomaterials and biomaterials, which in conjunction with the highly antigenic regions and/or subunits of the SARS-CoV-2 virus (S, N, or both), improved the affinity of the antibodies and/or increased the visible signal of antigen–antibody binding [94]. The main LFIA models for the detection of antibodies against SARS-CoV-2 have been designed in a competitive and sandwich type with antibodies coupled to colloidal gold nanoparticles (AuNPs), QDs, and fluorescent nanomaterials (FND) as visual reporters.

The LFIAs that use N (N-LFIA) viral antigen to detect IgG antibodies against SARS-CoV-2 contribute to identifying natural COVID-19 illness individuals that, once infected, produced high levels of neutralizing antibodies against N viral antigen up to two months after the onset of symptoms [95,96]. This time could be considered a diagnostic limit for the use of N-LFIA. Even it is possible that N-LFIA could detect SARS-CoV-2 in asymptomatic personnel at high risk. Nickel and colleagues (2022) carried out the N-LFIA serological test on at-risk healthcare workers and detected antibodies to the N viral protein with a sensitivity of >99%, indicating prior infection [86]. The early identification of individuals with antibodies against viral protein N could be considered a biomarker of the previous infection. The condition of the previous infection gives them an advantage from the first dose of vaccine (tested with Pfizer/BioNTech); they form antibodies against viral protein S, 2.7 times more than those not exposed to the virus [96]. Identifying anti-N antibody-negative individuals means there is no prior SARS-CoV-2 infection, so they will require a second dose of vaccine (Pfizer/BioNTech) to produce neutralizing antibodies against the antigenic S protein [96]. It has been proposed that in countries where vaccination against SARS-CoV-2 is of low availability, the N-LFIAs that detect previous SARS-CoV-2 infected individuals allow identifying naturally immunized individuals who could receive the vaccine some weeks later [97].

**Figure 3:**
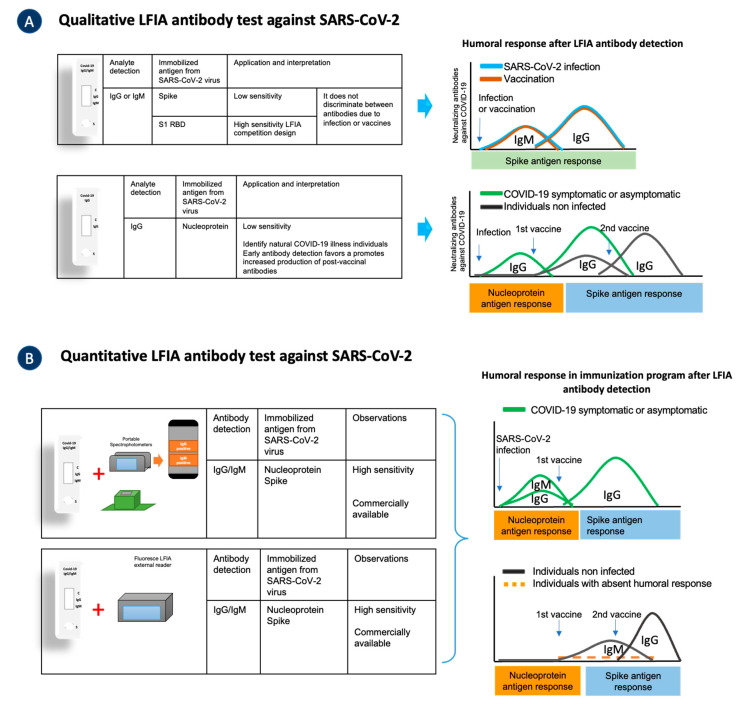
LFIAs detect IgG and/or IgM antibodies in asymptomatic, symptomatic, and immunized individuals. (**A**) LFIAs with qualitative readouts are available with the SARS-CoV-2 NP antigenic protein and the S protein. (**B**) LFIAs coupled to portable spectrophotometer have a greater sensitivity for evaluating the humoral response of convalescent, symptomatic, asymptomatic, and immunized individuals. The graphs on the right indicate the interpretation of LFIA results showing the humoral response in SARS-CoV-2 infected and uninfected individuals and those who have received at least the first dose of vaccine.

In regions where other beta-coronaviruses are present, their N-terminal domain of the N antigen is highly conserved, which may cause N-LFIAs to give false-positive results and/or fail to detect true early sensitization [98]. LFIA targeting the S antigens does not distinguish between vaccine- and infection-induced antibodies [99] (Figure 3A). The incorporation of the ACE2 receptor as an immobilized protein in the test pad assay line enhanced the LFIA sensitivity. This improvement favored an increased detection of the serum antibody complex with the receptor binding domain (RBD) of the S1 subunit of the SARS-CoV-2 virus. If a neutralizing antibody were present, it would bind to AuNP-RBD and prevent the AuNP-RBD from being captured by the immobilized ACE2 protein, indicating the absence of a tag in the test line [100].

A qualitative positive LFIA result is when the test line region is observed with low to marked color intensity. If the test line is not marked, the result is declared negative. The test is only valid if the control line shows a visible color in all cases. In some cases, it is difficult to interpret the color band by the naked eye when the intensity is low [101]. The strategy to increase the sensitivity of LFIA to detect IgG/IgM against SARS-CoV-2 and a positive cut-off threshold limit has been to transform the qualitative reading with the naked eye for a quantitative measure with the help of a portable spectrophotometer [94,101,102]. It was also observed that depending on the format in which the LFIA was manufactured (sandwich or competition type) and the viral antigen immobilized in the conjugation pad region, the test’s sensitivity could be improved. Chen and colleagues (2021) designed a combined LFIA with N and S antigens on the solid phase (conjugate pad) to recognize IgM/IgG from the serum sample. After antigen–antibody binding occurs, the complex migrates to the test line. A second binding is conjugated with colloidal gold-labeled anti-human IgM/IgG antibodies, evidencing the positive mark that a spectrophotometer quantifies. This LFIA coupled to a portable spectrophotometer detects a positive cut-off threshold of 0.5 ng/mL compared to 5–10 ng/mL detected by a qualitative LFIA. The LFIA spectrum analyzer uses a primary reflectance wavelength of 540–470 nm for samples with a low IgG concentration and a wavelength of 650 nm to reference IgG concentrations. The ratio between both wavelengths obtains the α value. A higher α value indicates a stronger reflection color intensity of the colloidal gold antibody-conjugated IgG and IgM complexes [94]. Hung and colleagues (2021) used an LFIA, with N protein as antigen in the conjugated pad, and anti-human IgG/IgM antibodies in the test line conjugate region [102]. The measure was performed using the same spectrophotometer as Chen and colleagues (2021) [94]. The authors reported an increase in the antibody detection limit to 186 pg/mL on these assays [102].

Chen and colleagues (2021) replaced colloidal gold with gap-enhanced Raman nanotags (GERTs) in an LFIA to detection of human IgM and IgG against SARS-CoV-2 recombinant antigens. The sensitivity was 1 ng/mL and 0.1 ng/mL for detection of IgM and IgG respectively [103]. Furthermore, in another study, Huang and colleagues (2022) included an LFIA with a competitive format, using the S antigen to detect IgG/IgM antibodies, a final capture in the test line region with the ACE-2 receptor, and the quantitative analysis was performed with a spectrophotometer [101]. The authors changed the antigen to detect neutralizing IgG/IgM antibodies in individuals vaccinated with the AstraZeneca COVID-19 vaccine. Moreover, they demonstrated that LFIA had efficient sensitivity and sensitivity as the ELISA assay (80 and 100%, respectively), with a Rho coefficient of 0.933. The LFIAs for detecting antibodies described here still require evaluating their efficacy with the new variants of the SARS-CoV-2 virus [101]. Pieri and colleagues (2022) used a kit that includes the LFIA sandwich-type (Affimedix Inc., Hayward, USA) that detects qualitative IgG neutralizing antibodies that recognize the S protein receptor-binding domain antigen. The cassette is placed in a RapidRead reader system (Affimedix Inc., Hayward, USA) for quantitative diagnosis of COVID-19 antibodies. The kit LFIA and reflective optical density reader have equal sensitivity to two chemiluminescence immunoassay methods [83].

The fluorescence LFIA (FLFIA) with a quantitative reading by a spectrofluorometer has high sensitivity to detect total antibodies. The evaluation of an FLFIA test with sandwich immunodetection method has included the N antigenic recombinant protein or S-RBD proteins as immobilized antigens. Both showed a high sensitivity (92–98.68%) to detect serum antibodies in SARS-CoV-2 symptomatic and asymptomatic patients. The authors recommend that these commercial tests assess binding and neutralizing antibody response after SARS-CoV-2 infection or vaccination (Figure 3B) [104,105].

Novel labeling nanomaterials can enhance fluorescence as a signal amplification label, improving the sensitivity of LFIA for detections of antibodies against SARS-CoV-2 antigens. Lanthanide-doped polystyrene nanoparticles (LNPs) can bind to mouse anti-human IgG antibodies, which are located on a conjugate pad as a fluorescent reporter. The readout was performed with a fluorescent reader, and the detection took 10 min. The test’s sensitivity was comparable to RT-PCR in 51 human serum samples [106].

Quantum dots nanobeads (QBs) made with CdSe/ZnS can be coated with Octadecylamine, which gives them highly luminescent properties. These QBs linked to the recombinant SARS-CoV-2 spike protein are placed on the conjugated pad where antibodies in the serum can recognize them. In experimental conditions, the QB-LFIA has high sensitivity with positive samples up to 10 times higher than a traditional AuNP-LFIA. The QB-LFIA showed a sensitivity of 97.1% and a specificity of 100% in assays with serum samples, although analysis with whole blood is required [107].

All quantitative LFIAs require a portable device as a reader, which could compromise their POC status. On the other hand, the amount of reagents required is considerably less than with traditional LFIAs, which would justify the cost/benefit for mass implementation.

Moreover, the humoral immune response against SARS-CoV-2 can generate null or high antibody production, which must be considered for any antibody test. It is recommended that the results of LFIA be complemented with RT-PCR for greater sensitivity in the diagnosis of SARS-CoV-2, as the detection of IgM/IgG antibodies by ELISA has been shown to have higher sensitivity when combined with RT-PCR (98.6%) than RT-PCR alone (92.2%) [108].

### 5.2. Lateral Flow Immunoassay Viral Antigen Testing for COVID-19

In 2020, the manufacture of LFIAs that detect SARS-CoV-2 specific viral structures (SARS-CoV-2 protein antigens N and S) increased because COVID-19 infection remains present in the vaccinated population worldwide. At the pandemic’s start, LFIA devices that detect the SARS-CoV-2 viral antigen were discarded and not recommended due to their limited reliability in patients with low viral load [108,109]. In regions of the world with a disease prevalence of less than 0.5%, antigen-based LFIAs were not recommended due asymptomatic individuals showed a positive predictive value of 11% to 28%, meaning that 7 out of 10 to 9 out of 10 positive results will be false positives, and 1 out of 2 to 1 out of 3 cases will be missed [109]. By increasing the spread of SARS-CoV-2 and turning it into a pandemic, the LFIAs were redesigned for the detection of SARS-CoV-2 antigens, highlighting that the S protein possessed the antigenicity that gave a remarkable improvement in the sensitivity and specificity of the test, although still below the standard test accepted worldwide, RT-PCR [104].

Improvements in LFIA testing led to clinical trials in Europe, Asia, and the USA, using various LFIA devices that showed high specificity (95–100%) while sensitivity was low (30–78%) [110]. The factors involved in the low sensitivity of the LFIAs tests that detect SARS-CoV-2 antigens were the time of infection, age, inadequate collection, sample conservation, and the quality of the product by the manufacturer. It has been determined that the time to detect the viral antigen with LFIA is in the first 7 days the patient develops symptoms because the highest viral load occurs in this period. Individuals with mild or asymptomatic symptoms or who exceed this time could have a low viral load [111]. In this disease period, LFIAs detect at least a Ct value < 25 (~100,000 RNA copies/mL), which is sufficiently accurate and helpful for mass population screening programs [112,113]. The WHO recommends using LFIAs, which meet the minimum performance requirements of ≥80% sensitivity and ≥97% specificity. To detect cases in symptomatic people suspected of being infected and asymptomatic people at high risk of COVID-19; for contact tracing; during outbreak investigations; and to monitor trends in disease incidence in communities [114]. The LFIAs are designed to take a nasopharyngeal swab sample. However, some researchers switched to saliva to reduce the risks of infection for the sampler. Saliva may affect the test due to the sample collection and storage technique, which could have implications for the low sensitivity of the assay [115,116]. These conditions have not yet been analyzed.

**Figure 4:**
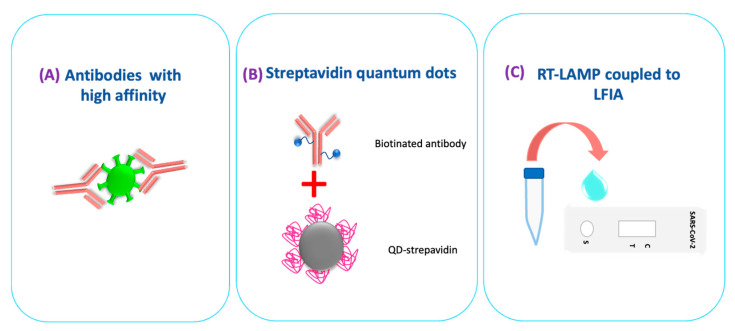
Strategies to improve the detection of SARS-CoV-2 antigens or nucleic acids by Lateral flow immunoassay. (**A**) The sensitivity of LFIAs focuses on developing high-affinity antibodies for the antigen. (**B**) Use of nanomaterials as markers that potentiate the antigen–antibody signal. (**C**) The detection of nucleic acids, incorporating different isothermal amplification techniques.

The main LFIA models for detecting SARS-CoV-2 antigen were competitive and sandwich types with antibodies coupled to AuNPs, quantum dots (QDs), and fluorescent nanodiamond (FND) as a visual reporter [117,118]. Afterward, LFIAs that detect viral antigens in the detection of SARS-CoV-2 focused the improvement of sensitivity on increasing the specificity of antibodies immobilized in the test, using nanomaterials as labels that increase the signal generated by antigen–antibody binding, and detection of nucleic acids (Figure 4).

The classical technology to produce monoclonal antibodies incorporated into LFIAs is insufficient to improve sensitivity against SARS-CoV-2 target proteins. Kim and colleagues (2021) resolved antibody affinity enhancement using phage display technology to develop an LFIA-based biosensor specific for SARS-CoV-2. Researchers generate phage-engineered monoclonal antibodies against SARS-CoV-2 NP. Newly developed antibodies specific for SARS-CoV-2 were conjugated to nanobead, with a sensitivity of 2 ng antigen protein and 2.5 × 10^4^ pfu cultured virus. The new LFIA platform gives an outcome that can be confirmed with the naked eye in 20 min and has a sensitivity of 100%, detecting only SARS-CoV-2 NP, not NPs from MERS-CoV, SARS-CoV, or influenza H1N1 [17]. Another advantage of this new LFIA is that the outcome can be analyzed and quantified using a portable LFIA reader. The authors tested the diagnostic device in experimental laboratory conditions, and non-human samples were tested [17]

Concerning labels that amplify the antigen–antibody binding signal, copper coupled to antibodies has been used to amplify the chromatographic signal for a minimum detection limit in the sample of 10 pg/mL [119].

The fluorescence immunochromatographic (FIC) method uses antibodies conjugated to fluorochromes to detect SARS-CoV-2 viral antigens. This test requires an immunofluorescence analyzer for the quantitative results. Diao and colleagues (2021) developed an FIC with mouse polyclonal antibodies and labeled them with fluorescent europium microparticles to detect the antigenic protein N of SARS-CoV-2. The authors reported a sensitivity of 75.6% [120]. In India, using a commercially available FIC (Standard F, SD Biosensor) made with monoclonal antibodies did not improve the sensitivity and was reported to be 38% [121]. The FIC assay is slower and less convenient than LFIAs and requires a battery-powered immunofluorescence analyzer. Incubation time can be up to 30 min, which affects test performance at the POC. Europium, used in ICF, has a fluorescence stability limit. It has been established that the time limit for performing the hybridization process is 30 min. After this time, a positive sample by RT-PCR will give a false positive. The limit to the correct detection of positive and negative reference samples is 15 min [122].

The fluorescent signal can be amplified by magnetic QD tags (Fe_3_O_4_-QD) (MagTQD). When this nanomaterial is used to amplify fluorescence in an LFIA strip, the biosensor could simultaneously detect SARS-CoV-2 S/NP antigens with high sensitivity (0.5–1 pg/mL). The biosensor is not yet tested with clinical samples [123]. Interestingly, high-affinity peptides have been developed that detect the RBD protein of the SARS-CoV-2 virus at levels as low as 0.01 nM. These peptides are inexpensive and easy to synthesize and could be coupled to LFIA to improve their sensitivity and reduce costs [124].

### 5.3. LFIA Simultaneously Detects Antigens and Antibodies

The biotechnology applied at LFIA has succeeded in developing a biosensor that simultaneously detects the alpha and beta antigenic variants of SARS-CoV-2 and the neutralizing antibodies. This device uses an immobilized ACE2 receptor to detect antigenic variants of the SARS-CoV-2 S protein from the nasopharyngeal sample via color differences of substrates. Antibody detection is achieved by a competition LFIA, where a sample containing neutralizing antibodies binds to the RBD antigen blocking the binding to the ACE2 receptor; thus *t* (test) line is absent [125].

## 6. LFIA Detects Nucleic Acids of SARS-CoV-2

The sensitivity of LFIAs remained questionable because, unlike RT-PCR, they could not be designed to detect SARS-CoV-2 nucleic acid (NA). Interestingly, advances in biotechnology have made it possible for LFIAs to integrate new methods of NA detection. The main methods for this detection are new high-affinity monoclonal antibodies and coupling isothermal amplification methods (IAM), such as RT-PCR and LAMP.

The method called hybrid capture fluorescence immunoassay (HC-FIA) uses S9.6 monoclonal antibodies, which were used to capture the hybridization of nucleic acids (SARS-CoV-2 DNA and RNA probes) on a lateral flow strip and immunofluorescence analysis. The HC-FIA test showed 100% sensitivity and 99% specificity from throat swabs and sputum samples [122].

Avidin-carbon nanoparticles (CNPs) have been evaluated as a label in LFIA for NA detection. This method increases the contrast in the paper background after the nucleic acid-antibody complex union. The visual limit of detection is in the nanomolar range (2.2 × 10^−2^ pg μL^−1^) without the assistance of any instrumentation. The advantages of CNPs are they are cheaper labels; the suspension is very stable and easy to modify. The critical point of this method is the double tagging of primers in the PCR procedure, which allows the posterior binding with Av-CNPs [126].

The IAM method tests Accula SARS-CoV-2 of Mesa Biotech combined RT-PCR and LFIA. The test cassette contains all reaction reagents and targets the N gene of SARS-CoV-2 from nasal and throat samples in 30 min, and the results are interpreted visually. The analytical sensitivity reported is 200 copies/mL (Mesa Biotech Inc., San Diego, USA, 2020). The clinical performance with 30 positive and 30 negative samples showed clinical sensitivity and specificity of 100% [3]. The disadvantage of this test is that it is limited to laboratories certified in EUA under the Clinical Laboratory Improvement Amendments of 1988 (CLIA). Still, where available, the test is considered POC (Mesa Biotech Inc., San Diego, USA, 2020). The methodology of combining LFIA and PCR implied a breakthrough in the fusion of reagents and biomaterials of both techniques. However, the complexity of manufacturing increased the risks of kit contamination. On 6 April 2022, Mesa Biotech, Inc. announced the recall of Accula SARS-CoV-2 tests due to possible contamination of the kit causing false positives in the individuals to which they are applied [127]. Agarwal and colleagues (2022) developed an LFIA that detects antigenic protein N in cDNA by combining RT-LAMP methodology [128]. In SARS-CoV-2 positive patients, the test demonstrated an accuracy of 81.66% and a minimum viral RNA detection limit of Ct < 33. The result of RT-LAMP in combination with LFA can be completed with a smartphone-based semi-quantitative data analysis [128]. The challenges of LFIA for nucleic acid detection are that isothermal amplification requires a complex primer design, and the signal can only be analyzed by portable readers [129,130].

## 7. Conclusions and Perspectives

LFIAs have been a very efficient tool during the pandemic due to their simplicity, speed, and cost-effectiveness. LFIAs can be used in low-resource field settings and in developing countries that cannot use other methods for SARS-CoV-2 detection. However, there are still some challenges to face. One of the main challenges is to develop their ultra-sensitivity, together with POC advantages, especially in samples that require more than one step to detect the virus. The improvement in LFIAs includes: (1) the synthesis of immobilized biomaterials, antigens, or antibodies in the conjugation region with high affinity to the analyte; (2) the use of new nanomaterials to label the antigen or antibody that is conjugated to the analyte; (3) quantitative analysis using an external device. It is important to highlight that current LFIAs’ performances are comparable with ELISA and chemiluminescence immunoassay methods. The results can be quantitative and qualitative according to the epidemiological needs or the vaccination present in the region or country. In addition, the quantitative LFIAs could contribute to data digitization, allowing the monitoring, storage, and transmission of data, reducing interpretation and transcription errors, and thus ensuring the quality and control of the tests. LFIAs are highly adaptable devices. In the future, it has been proposed that LFIA will focus on detecting SARS-CoV-2 virus nucleic acids by incorporating isothermal amplification methods under non-laboratory conditions and novel labeling materials that amplify the signal. The implementation of low-cost handheld readers, including smartphones, will be able to match or even improve the efficiency of LFIA on par with the RT-PCR test.

## Data Availability

Not applicable.
